# AtOM, an ontology model to standardize use of brain atlases in tools, workflows, and data infrastructures

**DOI:** 10.1038/s41597-023-02389-4

**Published:** 2023-07-26

**Authors:** Heidi Kleven, Thomas H. Gillespie, Lyuba Zehl, Timo Dickscheid, Jan G. Bjaalie, Maryann E. Martone, Trygve B. Leergaard

**Affiliations:** 1 Department of Molecular Medicine, Institute of Basic Medical Sciences, University of Oslo, Oslo, Norway; 2 Department of Neurosciences, University of California, San Diego, USA; 3 Institute of Neuroscience and Medicine (INM-1), Research Centre Jülich, Jülich, Germany; 4 Institute of Computer Science, Heinrich Heine University Düsseldorf, Düsseldorf, Germany

**Keywords:** Computational neuroscience, Brain

## Abstract

Brain atlases are important reference resources for accurate anatomical description of neuroscience data. Open access, three-dimensional atlases serve as spatial frameworks for integrating experimental data and defining regions-of-interest in analytic workflows. However, naming conventions, parcellation criteria, area definitions, and underlying mapping methodologies differ considerably between atlases and across atlas versions. This lack of standardized description impedes use of atlases in analytic tools and registration of data to different atlases. To establish a machine-readable standard for representing brain atlases, we identified four fundamental atlas elements, defined their relations, and created an ontology model. Here we present our Atlas Ontology Model (AtOM) and exemplify its use by applying it to mouse, rat, and human brain atlases. We discuss how AtOM can facilitate atlas interoperability and data integration, thereby increasing compliance with the FAIR guiding principles. AtOM provides a standardized framework for communication and use of brain atlases to create, use, and refer to specific atlas elements and versions. We argue that AtOM will accelerate analysis, sharing, and reuse of neuroscience data.

## Introduction

Brain atlases are essential anatomical reference resources that are widely used for planning experimental work, interpreting and analyzing neuroscience data^1–12^. Three-dimensional (3D) digital brain atlases^3,13–17^ are increasingly employed as frameworks for integrating, comparing, and analyzing data based on atlas-defined anatomical locations (e.g. Allen brain map (https://portal.brain-map.org); the BRAIN Initiative Cell Census Network (https://www.biccn.org); the EBRAINS research infrastructure (https://ebrains.eu)). These resources provide anatomical context suitable for brain-wide or region specific analysis using automated tools and workflows^18–26^ and facilitate sharing and using data in accordance with the FAIR principles^27^, stating that data should be findable, accessible, interoperable, and reusable. However, the use and incorporation of different atlas resources in such workflows and infrastructures requires that atlases, tools, and data are interoperable, with relatively seamless exchange of standardized machine-readable information.

Most brain atlases share a set of common properties, but the specifications and documentation of their parts differ considerably. Detailed versioning is not yet common practice for all atlases and lack of specific information about changes in the terminology or anatomical parcellation make it difficult to compare atlas versions. While some gold standards have been established^28^, lack of consensus regarding the presentation, specification, and documentation of atlas contents hampers reproducible communication of locations^11^ and comparison of data that have been anatomically specified using different atlases^10,24^. Atlases and their versions need to be uniquely identifiable and interoperable to enable researchers to communicate specific and reproducible location data and integrate data across specialized neuroscience fields and modalities.

To address the lack of standardization of atlas metadata, we identified four common atlas elements, defined their relations, and created the Atlas Ontology Model (AtOM). By specifying the relations and hierarchies of objects and processes in an ontology model^29^, we created systematic and coherent links among data files, metadata, and process descriptions enabling automated retrieval of information in using computational tools^30^.

Here we characterize the properties and relations of the elements of brain atlases and explain their organization in the model. Using the relations defined by AtOM we show that any specific set of atlas elements and their associated metadata makes up a unique version of an atlas. Furthermore, we suggest a set of minimum requirements for atlases inspired by the FAIR principles and discuss how atlases adhering to AtOM could accelerate neuroscience data integration.

## Results

We investigated a broad selection of mammalian brain atlases^3,13,14,17,31–39^ and identified four common elements: (1) a set of reference data, (2) a coordinate system, (3) a set of annotations and (4) a terminology. Below, we describe these atlas elements and their relations, and exemplify how they can be identified in different atlases. We go on to show how the ontology model allows specification of unique atlas versions. Lastly, we employ AtOM to suggest minimum requirements for FAIR brain atlases and briefly describe how these requirements facilitate the incorporation of brain atlases into research workflows and software tools.

### The atlas elements

**Fig. 1:**
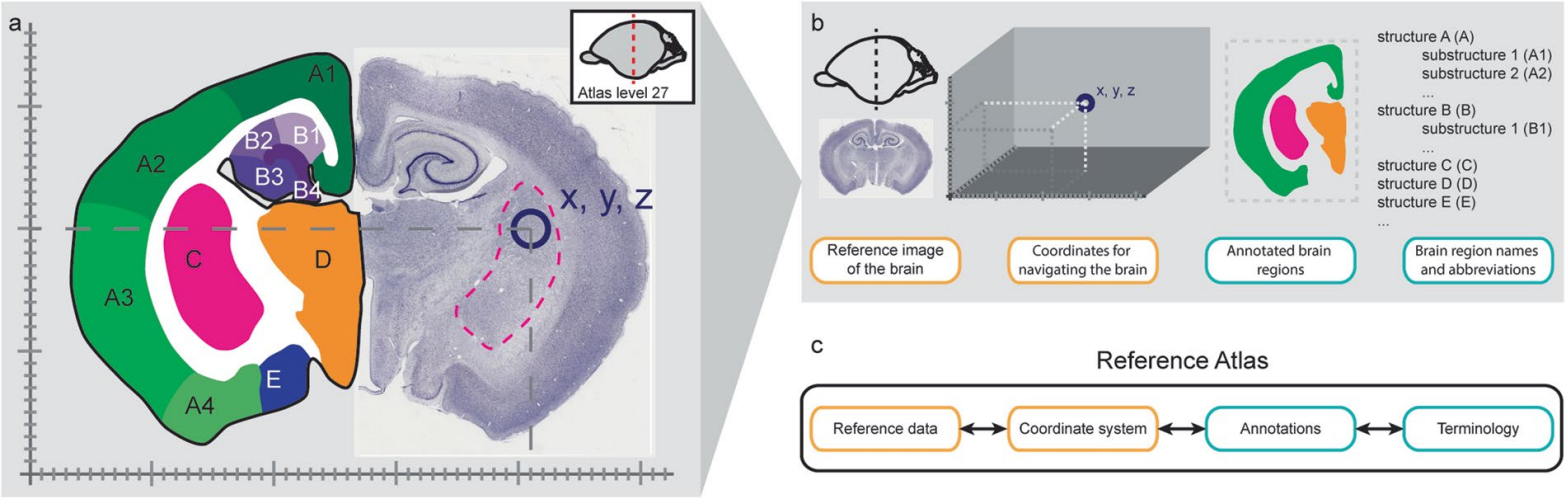
Atlas Ontology Model elements. (**a-b**) The elements of a fictional two-dimensional brain atlas illustrated using a coronal Nissl-stained section and a drawing of the Platypus (*ornithorhynchus anatinus*) brain^79^. (**c**) The Atlas Ontology Model, formalizing the elements of a reference atlas.

**Fig. 2:**
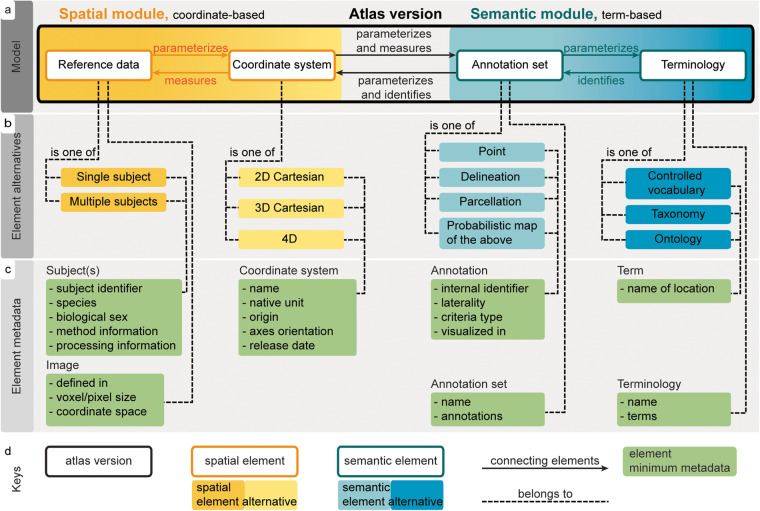
The relations and metadata of the AtOM elements. (**a**) Diagram illustrating the relations between the AtOM elements: *measures* (to provide a metric to)*, parameterizes* (to set the conditions of its operation) and *identifies* (to recognize, establish or verify the identity of something). Thus, the coordinate system measures the reference data and annotation set with coordinates as units. The terminology identifies the annotation set and coordinate system with terms as units. The reference data and the annotation set provide physical dimensions embodying the coordinate system and the terminology. The model consists of two reference modules: *spatial* (containing the coordinate system and reference data, yellow) and *semantic* (containing annotations and terminology, blue). Each element can be one of a set of alternatives (**b**), which have a set of metadata (**c**). (**d**) Key for reading the figure.

The atlas elements in AtOM are the reference data, coordinate system, annotation set, and terminology (Figs. 1, 2). Each of the four elements have properties, such as identifier, species, sex, and age, specified with detailed metadata (Fig. 2b,c).

The *reference data* of a brain atlas are graphical representations of one or several brains, or parts of brains, chosen as the biological reference for that atlas. The reference data typically consist of histological or tomographic images. These images may be derived from a selected specimen, with the assumption that it represents generalizable biological features within its age category and biological sex. This is the case for the BigBrain human brain atlas (with reference data showing cytoarchitecture of one adult male^16^), and for many rodent atlases (which typically use reference data from a single adult male of a certain strain, e.g. Sprague Dawley^14,35^ or Wistar^34^, Fig. 2b). Alternatively, some atlases use reference data compiled from several subjects representing different features or image orientations, e.g. several rat brains cut in one or all three standard orthogonal planes^37,40^. Reference data may also be acquired by averaging data across many subjects, i.e. by creating a population average constructed from spatially co-registered images^17^. An example of this is the Allen Mouse Brain Atlas Common Coordinate Framework (AMBA CCF)^3,13^, generated by averaging 1675 mouse brains acquired by serial two-photon microscopy. The spatial resolution of the reference data determines the level of detail that can be identified. For example, the widely adopted human reference datasets of the Montreal Neurological Institute (MNI)^41,42^ are based on averaged magnetic resonance imaging (MRI) scans and represent suitable reference data for macroscopic anatomy, while the single-subject *BigBrain* model^33^ provides a reference dataset for identification of cortical layers and more fine-grained cortical and subcortical structures^16^.

The *coordinate system* of an atlas provides a framework for specifying locations with origin, units and direction of the axes^43^ (Fig. 2c). In brain atlases, the coordinate system origin is often defined using a characteristic feature of the skull, e.g. the *bregma* in a stereotaxic coordinate system^34,35^, or a specific anatomical landmark identified within the brain, e.g. the decussation of the anterior commissure in the Talaraich-Tournoux space^44^ and Waxholm Space coordinate system^14,45^. The orientation is given by the direction of the axes. For example, the axes of AMBA CCF are directed towards posterior (P), inferior (I) and right (R), giving the orientation PIR (http://help.brain-map.org/display/mousebrain/API). The coordinate system is usually, but not always, a 3D Cartesian coordinate system. Examples of coordinate systems which go beyond a 3D Cartesian system include spatio-temporal systems, with additional time or surface dimensions^46^.

The *annotation set* of an atlas consist of graphical marks or labels referring to spatial locations determined by features observed in, inferred from, or mapped onto the reference data, specifying structures or boundaries. An annotation set may identify features-of-interest as points, for example by placing a name or abbreviation on the area of a brain region. Although such annotations can give the user an overview of prominent landmarks and regions in the brain, they are limited in that they do not define the borders of the regions. Thus, most book atlases^34,35^ demarcate anatomical boundaries or regions with lines, while 3D brain atlases such as the AMBA CCF v3^3^ or WHS rat brain atlas v4^14,47^ fully delineate regions with closed curves. In the case of probabilistic maps, coordinates are labeled with the probabilities of a certain region or feature being present at a given location^17,48–50^. Probabilistic maps are typically aggregated from annotations identified in different individuals, encoding variation across subjects^17^. To summarize, an annotation set can consist of points, lines or closed curves, or probabilistic representations of any of these (Fig. 2b).

The *terminology* of an atlas is a set of terms that identifies the annotations, providing human readability and context, and allowing communication about brain locations and structural properties. In its simplest form, a terminology can be a list of unique identifiers, but is typically a set of descriptive anatomical terms following specific conventions. Atlases employ different terms, conventions, and approaches to organize brain structures into systems based on the methodology used to create them as well as their intended use cases. For example, some use developmental organization^51,52^, while others use brain systems^39^, microstructural organization^16^, multimodal features^53^, or are specialized for particular brain regions^54,55^. An atlas terminology may be a controlled vocabulary (flat list, e.g. the label file of the Waxholm Space atlas of the Sprague Dawley rat brain), a taxonomy and partonomy (hierarchical list, e.g. the Allen Mouse Reference Atlas Ontology (RRID:SCR_021000)), or an ontology (hierarchy and additional axioms, e.g. that two structures are adjacent).

### Relations among the elements

The four elements of AtOM have specific relations (specified in Fig. 2a), sorted into a *spatial module*, consisting of the reference data and the coordinate system (Fig. 2a, yellow), and a *semantic module*, consisting of the annotation set and the terminology (Fig. 2a, blue).

The elements of the *spatial module* provide the physical and measurable dimensions of the atlas. The biological dimensions of the reference data give the conditions of operation for (i.e., *parameterize*) the coordinate system. The coordinate system provides a metric for (i.e., *measures*) the reference data, specifying the origin, orientation, and units (Fig. 2a). Coordinates are the means to derive measurements, indicate directions, and spatially locate features in the reference data. The coordinate system also *measures* the annotation set, and thus connects the annotations to the features of the reference data. The two spatial elements can be intricately linked, for example through the process of generating the reference data based on multiple subjects. Knowing the detailed information about these links or processing dependencies is not necessarily needed for using an atlas version. However, it is often very useful to have as much metadata and documentation as possible to understand how the two elements are related to each other, especially if one of the elements is changed or when comparing two different atlases to translate information between them.

The elements of the *semantic module* provide semantic identities for the atlas. The annotation set *parameterizes* the terminology in the spatial domain according to or inspired by the reference data. The terminology provides terms to establish the identity of (i.e., *identifies*) each annotation (Fig. 2a). While anatomical terms are not unique identifiers (see Atlas versioning below), they provide a means to semantically address annotations, conveying neuroanatomical knowledge and context. In this way, the terms are semantic units suitable for navigating the atlas annotations, while annotations capture the scholarly interpretations and knowledge underlying the experimental and anatomical criteria used to make them (parcellation criteria). Further, the annotation set propagates the semantic identities from the terminology, and thus semantically *identifies* locations in the coordinate system. The semantic elements may also be linked through the criteria for defining the extent of an annotation, which is often summarized in the name and thus in the terminology. Again, this information is not essential for using an atlas version, but critical for translating information across elements.

The relations of the atlas elements are pathways for translating information between the spatial and semantic modules. A researcher may consult an atlas to observe the physical shape and location associated with a given anatomical term, or to identify the anatomical term assigned to specific coordinates, or biological features observed in the reference data. Thus, the model is a continuous, bidirectional loop providing several starting points for researchers to translate and compare information across atlas elements.

### Using AtOM to identify elements in brain atlases and communicate location

AtOM is also readily applied to traditional stereotaxic book atlases^34,35,56–58^ as illustrated in the fictive brain atlas in Fig. 1. In principle, a brain atlas can be a set of images with names indicating areas, coordinates for each histological image, and orientation indicators. While the precision of such an atlas might be limited, it can still be versioned and used to communicate reproducible information about brain location.

**Fig. 3:**
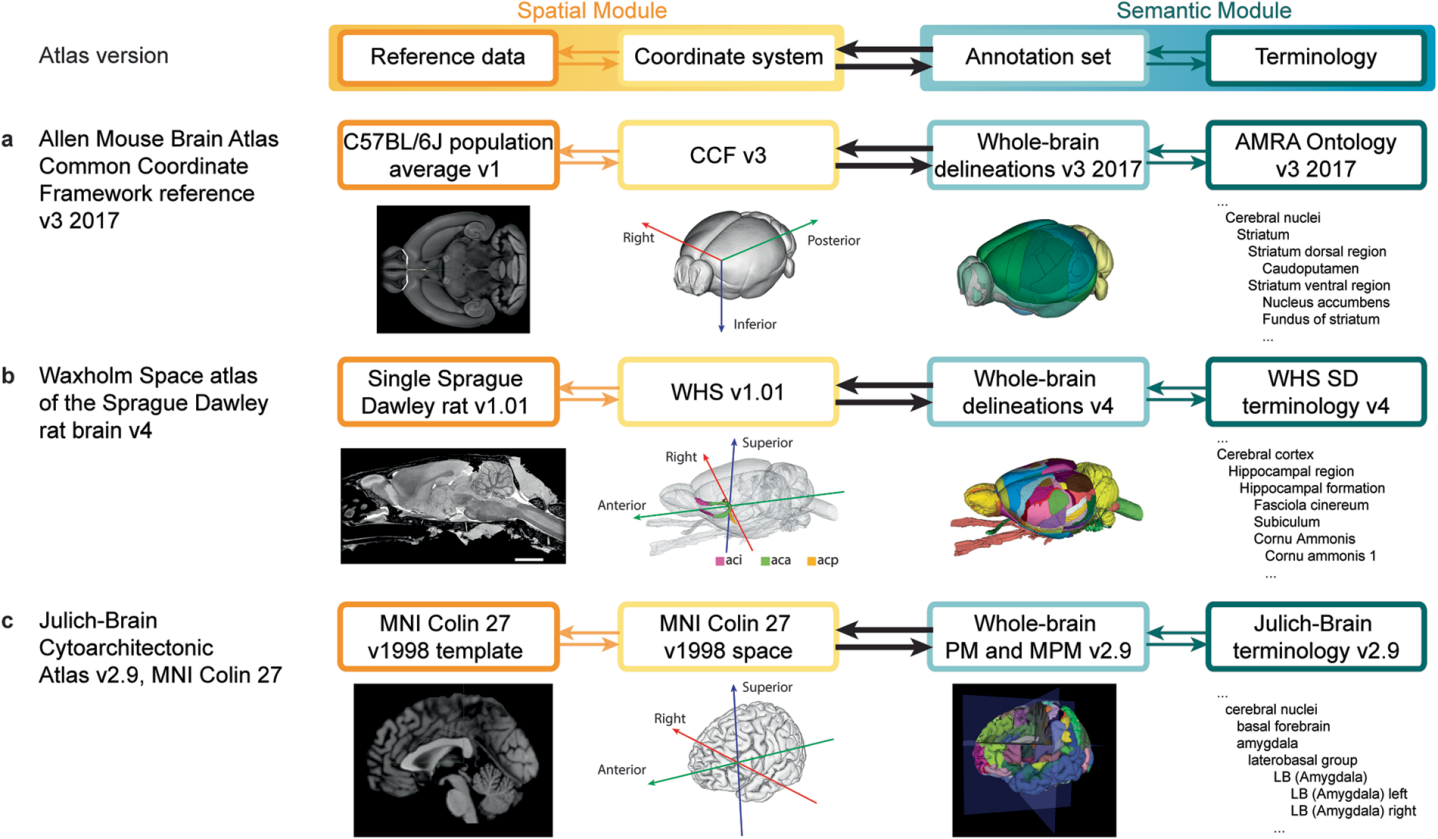
AtOM elements illustrated for three brain atlas versions. (**a**–**c**) Tabular illustration of the most recent versions of (**a**) the Allen Mouse Brain Atlas Common Coordinate Framework^3^, (**b**) the Waxholm Space atlas of the Sprague Dawley rat brain^14^ and, (**c**) one alternative representation of the Julich-Brain cytoarchitectonic atlas^17^ organized in accordance with the AtOM diagram (top row). All atlases are accessible in the EBRAINS research infrastructure. Specification of the metadata, licenses and versions of these atlases are given in Tables 1, 2. CCF, Common Coordinate Framework; AMRA, Allen Mouse Reference Atlas; WHS, Waxholm Space; MNI, Montreal Neurological Institute; PM, probabilistic maps; MPM, maximum probability maps.

Figure 3 illustrates how AtOM can be used to identify elements and modules in 3D brain atlases. The reference data for the AMBA CCF v3 2017^3^ (Fig. 3a) consists of a population averaged serial two-photon tomography (STPT) volume created from 1,675 mice. The coordinate system is the CCF v3, which was created specifically for the Allen Institute mouse brain atlases. The annotation set is the whole-brain delineations from 2017, described in the accompanying white paper (http://help.brain-map.org/display/mouseconnectivity/Documentation), and the terminology is Allen Mouse Reference Atlas Ontology (RRID:SCR_021000). All the version specific metadata for the three atlas versions are listed in Table 1.

**Table 1:** Overview of metadata and licenses provided with mouse, rat and human brain atlas versions used in the EBRAINS research infrastructure.

Full name	Allen Mouse Brain Atlas Common Coordinate Framework v3 2017	Waxholm Space atlas of the Sprague Dawley rat brain v4	Julich-Brain Cytoarchitectonic Atlas v2.9, MNI Colin 27
Short name	AMBA CCF v3 2017	WHS rat brain atlas v4; WHSSDv4	Julich-Brain v2.9, Colin 27
Version identifier	3, 2017	4	2.9, Colin 27
Version innovation	Publication^3^; White paper AMBA CCF v3 2017 (http://help.brain-map.org/display/mouseconnectivity/Documentation)	Publication^14,47^; Webpage (https://www.nitrc.org/projects/whs-sd-atlas)	Publication^17^; EBRAINS datasets^59,60^
Alternative version of	NA	NA	Julich-Brain v2.9, MNI 152; Julich-Brain v2.9, BigBrain; Julich-Brain v2.9, fsaverage
New version of	AMBA CCF v3 2016	WHS rat brain atlas v3.01	Julich-Brain v2.5, Colin 27
Release date	NA	01.10.2021	31.07.2021
Reference data	C57BL/6 J population average v1	Sprague Dawley rat v1.01	MNI Colin27 v1998 template
Coordinate system	CCF v3	WHS v1.01	MNI Colin27 v1998 space
Annotation set	Whole-brain parcellation, v3 2017	Whole-brain parcellation, v4	Whole-brain probabilistic maps and maximum probability maps
Terminology	Allen Mouse Reference Atlas Ontology	WHS SD terminology, v4	Julich-Brain terminology, v2.9
License	Not available, but see legal note (https://alleninstitute.org/legal/citation-policy)	Creative Commons Attribution (CC BY) 4.0	Creative Commons Attribution-NonCommercial-ShareAlike (CC BY‐NC-SA) 4.0

AMBA, Allen Mouse Brain Atlas; CCF, Common Coordinate Framework; MNI, Montreal Neurological Institute; SD, Sprague Dawley; WHS, Waxholm Space.

### Atlas versioning

With an overview of the elements and relations of AtOM at hand, we are now in position to examine how they facilitate clear versioning of an atlas. In AtOM, an atlas version is a concrete instance of an atlas, and consists of specific elements, relations, and metadata (Fig. 2). Figure 3 and Table 1 show the metadata available for the most recent versions of the EBRAINS research infrastructure supported mouse^3^, rat^14^, and human^17^ brain atlases modeled using AtOM. An important consequence of AtOM is that the atlas version changes if there are alterations to any element. Examples of alterations include revising annotations or terms, modifying the reference data or coordinate system, or replacing an element. Such changes have consequences for the specific properties and use of an atlas and should be specified as a new atlas version. The changes made from one version to another can be described in atlas version documentation, and new versions of an atlas are usually distinguished by a new version name. The simplest way to do this is by iterative version numbering. Table 2 shows a complete overview of all versions of the AMBA CCF^3,13^, the Waxholm Space atlas of the Sprague Dawley rat brain (WHS rat brain atlas)^14,38,39,47^, and selected alternative versions of the Julich-Brain Cytoarchitectonic Atlas (Julich-Brain Atlas)^17^. In the last versions of the AMBA CCF (v3 2015–2017; http://help.brain-map.org/display/mousebrain/Documentation)^3,13,32^ and the WHS rat brain atlas (v1.01-v4)^14,31,38,39,47^ the semantic elements (annotation set and terminology) have been changed across versions, while the spatial elements (reference data and coordinate system, Table 2) have been kept constant. This continuation across versions allows translation of information and experimental data registered to the reference data are compatible with all versions of the mouse and rat atlas versions.

**Table 2:** Overview of the AtOM elements constituting the mouse, rat and human brain atlas versions currently supported by the EBRAINS research infrastructure.

Species	Version number	Atlas version name (semantic ID)	Reference data	Coordinate system	Annotation set	Terminology	Reference(s)
Mouse	1	Allen Mouse Brain Common Coordinate Framework reference atlas v1	C57BL/6 J population average v1	CCF v1	Whole-brain delineations v1	OWL AMBA terminology v1	RRID:SCR_020999; http://help.brain-map.org/display/mousebrain/Documentation^32^;
	2	Allen Mouse Brain Common Coordinate Framework reference atlas v2		CCF v2	Whole-brain delineations v2	Allen Mouse Reference Atlas Ontology	RRID:SCR_020999; RRID:SCR_021000; http://help.brain-map.org/display/mousebrain/Documentation^13^;
	3	Allen Mouse Brain Common Coordinate Framework reference atlas v3 2015		CCF v3	Whole-brain delineations v3 2015	Allen Mouse Reference Atlas Ontology	RRID:SCR_020999; RRID:SCR_021000; http://help.brain-map.org/display/mousebrain/Documentation^3^;
		Allen Mouse Brain Common Coordinate Framework reference atlas v3 2016			Whole-brain delineations v3 2016	Allen Mouse Reference Atlas Ontology	RRID:SCR_020999; RRID:SCR_021000^3^;
		Allen Mouse Brain Common Coordinate Framework reference atlas v3 2017			Whole-brain delineations v3 2017	Allen Mouse Reference Atlas Ontology	RRID:SCR_020999; RRID:SCR_021000; http://help.brain-map.org/display/mouseconnectivity/Documentation^3^;
Rat	1	Waxholm Space atlas of the Sprague Dawley rat brain v1	Single Sprague Dawley rat v1	WHS v1	Whole-brain delineations v1	WHS SD terminologyv1	RRID: SCR_017124; https://www.nitrc.org/projects/whs-sd-atlas^14^;
	1.01	Waxholm Space atlas of the Sprague Dawley rat brain v1.01	Single Sprague Dawley rat v1.01	WHS v1.01	Whole-brain delineations v1.01	WHS SD terminology v1.01	RRID: SCR_017124; https://www.nitrc.org/projects/whs-sd-atlas^31^;
	2	Waxholm Space atlas of the Sprague Dawley rat brain v2			Whole-brain delineations v2	WHS SD terminology v2	RRID: SCR_017124; https://www.nitrc.org/projects/whs-sd-atlas^38^;
	3	Waxholm Space atlas of the Sprague Dawley rat brain v3			Whole-brain delineations v3	WHS SD terminology v3	RRID: SCR_017124; https://www.nitrc.org/projects/whs-sd-atlas^39^;
	3.01	Waxholm Space atlas of the Sprague Dawley rat brain v3.01			Whole-brain delineations v3.01	WHS SD terminology v3.01	RRID: SCR_017124; https://www.nitrc.org/projects/whs-sd-atlas
	4	Waxholm Space atlas of the Sprague Dawley rat brain v4			Whole-brain delineations v4	WHS SD terminology v4	RRID: SCR_017124; https://www.nitrc.org/projects/whs-sd-atlas^14,47^;
Human^*^	1.18	Julich-Brain Cytoarchitectonic Atlas v1.18, MNI Colin 27	MNI Colin 27 v1998 template	MNI Colin 27 v1998 space	Whole-brain PM and MPM v1.18	Julich-Brain terminology v1.18	RRID:SCR_023277^78^;
		Julich-Brain Cytoarchitectonic Atlas v1.18, MNI 152	MNI ICBM 152 (2009c nonlin asym) template	MNI ICBM 152 (2009c nonlin asym) space			RRID:SCR_023277^78^;
		Julich-Brain Cytoarchitectonic Atlas v1.18, BigBrain	BigBrain (v2015) template	BigBrain (v2015) space	High-resolution maps v1.18		RRID:SCR_023277^33^;
	2.9	Julich-Brain Cytoarchitectonic Atlas v2.9, MNI Colin 27	MNI Colin 27 v1998 template	MNI Colin 27 v1998 space	Whole-brain PM and MPM v2.9	Julich-Brain terminology v2.9	RRID:SCR_023277^17,59,60^;
		Julich-Brain Cytoarchitectonic Atlas v2.9, MNI 152	MNI ICBM 152 (2009c nonlin asym) template	MNI ICBM 152 (2009c nonlin asym) space			RRID:SCR_023277^17,59,60^;
		Julich-Brain Cytoarchitectonic Atlas v2.9, BigBrain	BigBrain (v2015) template	BigBrain (v2015) space	High-resolution maps v2.9		RRID:SCR_023277^16,33^;
		Julich-Brain Cytoarchitectonic Atlas v2.9, fsaverage	fsaverage surface v1	fsaverage space v1	Surface projections v2.9		RRID:SCR_023277^17,61^;

*Only two major releases, each with their alternative versions (representations of the annotation set in different coordinate systems and respective reference data) of the human brain atlas are shown here.

To clearly reference a specific atlas version or AtOM element, it needs a unique identifier (ID). This is particularly important when combining different versions of elements into alternative atlas versions. The major release v2.9 of the Julich-Brain Atlas (Table 2) has four alternative versions due to its use of four complementary spatial modules: the “MNI Colin 27” (individual specimen, 1 mm resolution), “MNI 152” (population average, 1 mm resolution), “BigBrain” (individual specimen, 20 µm resolution) and “fsaverage” (cortical surface representation)^16,33,59–61^. These alternative versions are identified by combining the major release identifier (v2.9) with the abbreviated name of the respective reference data and coordinate systems. Unique identifiers are also important to differentiate between identical terms, which are often similar, but not identical, anatomical areas within and across species and atlases. Ambiguity can be avoided by indexing atlas version specific terms and providing unique ontology IDs defining their properties and relations. Following AtOM, an atlas version should have unique IDs for each element and their instances, which together with version documentation facilitate clear referencing of atlas versions and specific atlas elements.

### Minimum requirements for FAIR brain atlases

Atlases are a type of research data and thus can be evaluated using the foundational principles of the FAIR guidelines^27^. These principles state that data should be findable, accessible, interoperable, and reusable through both human and machine-driven activities. Like experimental data, atlases can support these principles through use of unique identifiers, specific metadata, open protocols, and clear usage licenses. Furthermore, interoperability and reuse of data also requires use of “formal, accessible, shared, and broadly applicable language for knowledge representation”, as well as metadata providing detailed descriptions. Based on our proposed ontology model, we suggest the following set of four minimum requirements for FAIR brain atlases: 1) machine readable digital components, 2) defined spatial and semantic modules with element metadata, 3) specification of element versions with detailed documentation, and 4) defined element relations and metadata (Fig. 1d,e). We elaborate on these requirements below.

First, *machine-readable digital atlas components* imply that all files and metadata are available in open and non-proprietary file formats suitable for direct processing by a machine. This enables programmatic access to critical information about brain atlases without the need to retrieve entire, potentially distributed, datasets. It also makes it possible to incorporate the information into research workflows and software tools, e.g. the siibra tools suite^62,63^ for exploring high-resolution atlases such as the multilevel framework established for the human brain (https://ebrains.eu/service/human-brain-atlas) and connecting them to computational workflows. The files and metadata for all the atlas versions shown in Fig. 3 are available online, either on public websites, domain repositories, or at the atlases’ respective homepages. Table 1 shows brain atlas version metadata for the four brain atlas versions shown in Fig. 3.

Second, *defined spatial and semantic modules* in an atlas mean that all elements are identifiable and accessible with clear metadata. This makes common elements between atlases or atlas version more comprehensible and facilitates the maintenance of atlases and their versions. At a minimum, this can be clear naming of the essential files or documentation about the location of all necessary information (Table 1). For example, all the files needed for using the WHS rat brain atlas are available via a domain repository (Table 2).

Third, *clear versioning with granular documentation* that state all changes differentiating two version of an atlas are needed to adhere to open science and FAIR principles. Currently this is partially achieved through use of persistent identifiers for atlas releases using either International Standard Book Numbers (ISBN), and Digital Object Identifiers (DOI) or Research Resource Identifiers (RRID)^64^. In addition, atlas reference data are made available as associated files^40^, as downloadable internet resources^3,16,17,39^, or by providing selected methodological descriptions in publications^14,17^. Some atlases also provide documentation as a list, or as text describing new features or a high-level inventory of changes. Ideally, clear versioning of all atlas elements would enable novice users to quickly identify the differences between two versions (Table 2).

Fourth, the *explicit relations between atlas elements*, such as parcellation criteria and coordinate system definitions, provide an empirical foundation for translating information across the elements. This allows users to connect data to different atlas elements (semantic or spatial), and enables automated search or comparison of data based on atlas elements. Traditionally, methodological information is mainly presented in a human-readable format through publications^14,17^, white papers or via a webpage, but it is now possible to document information in machine-readable, structured formats following standards, e.g. as single or distributed data publications^60^ (Table 2).

Brain atlases that fulfill these four requirements are thus expected to be sufficiently well defined to be incorporated into research workflows and enable automated transfer of information across atlases. The advantage of AtOM can be demonstrated with a concrete scenario where a researcher wants to create a modified version of an atlas to adapt the granularity of the brain annotations to their data. For example, in the following publication^65^ they used the hierarchical terminology to group selected brain regions of the AMBA CCF v3 2017 into larger custom regions and thus create a custom brain atlas version for their analysis. This was possible as the annotation set, terminology and metadata were readily available and identified (according to AtOM) and allowed the researchers to create and cite the changes in their custom atlas version. Another potential advantage of having individual atlas elements provided as separate files is that they may be used as exchangeable components in viewers or analysis tools such as siibra-explorer^63^ or siibra-python^62^. This allows for comparative analysis or re-analysis using different atlas versions^47^.

## Discussion

We have identified spatial and semantic elements of brain atlases, defined their relations, and created an Atlas Ontology Model (AtOM), specifying human and machine-readable metadata. Even though the AtOM elements are readily recognized in different atlases, they are often named according to traditions or common practice. For example, the reference data and the coordinate system are often considered as one entity, and referred to as the common coordinate space, reference template, reference space, brain model or atlas^9,42^. The term atlas is variably used to address reference data, an atlas version, any of a series of atlas versions or the annotation set. The annotation set, often in combination with the terminology, has also been called parcellations, segmentations or delineations^16,17,39,48^.

Some of the AtOM elements have been suggested earlier^9^, as well as similar approaches to versioning and atlas organization^17^. However, AtOM is the first model for standardizing the common elements of any brain reference atlas, their definitions, and metadata, creating a standard to organize and share information about atlases or as a template to create an atlas.

When implemented, AtOM will facilitate precise and unique referencing of parts of an atlas, as well as the incorporation of atlases in digital tools or workflows. AtOM further provides a basis for specifying minimum requirements for brain atlases to comply with the FAIR principles. Below, we discuss how AtOM may contribute to increase interoperability among atlases, enable more standardized use of brain atlases in computational tools, and advance FAIR data sharing in neuroscience. Interoperable atlases allow for exchange and translation of information across atlases, tools and data. Experimental data generated by different researchers typically relate to an atlas via spatial coordinates or anatomical terms, often defined by visual comparison of images or use of other observations such as measurements of functional properties. Researchers translate between the semantic and spatial location information using human readable metadata. At the same time, automated translation can be enabled via standardized, machine-readable files specifying properties and relations among atlas elements. The translation of information is dependent on interoperability across atlas elements, which can be specified at three levels: practical, technical, and scholarly.

At the *practical level*, translation of information across atlas elements is essential for interpretation and communication of anatomical locations, such as relating machine-readable coordinates to human-readable brain structure names. The relations specified between atlas elements and the defining metadata allow comparisons of annotations and terminologies across atlases representing different species or strains, developmental stages, or disease states. By aligning reference data or coordinate systems of two different atlases, information can be directly compared or translated. For example, aligned brain region annotations can be inspected and their respective terminology aligned, establishing a semantic translation across two atlas terminologies^66^. Alternatively, terminology and annotations from different atlases may be combined, as was demonstrated when creating a new unified mouse brain atlas by adopting the semantic elements from the Franklin and Paxinos mouse brain atlas into the AMBA CCF^67^. However, it is important to keep in mind that reproducible use of atlas resources depends on unambiguous citation of atlases and their versions. When the atlas version reference is ambiguous, or if anatomical names are given without specification of the employed atlas version terminology, it is difficult to compare location between datasets^11^. Versioning, documentation, and clear references are therefore essential for atlases that change over time.

At a *technical level*, atlas information can be accessed using computational tools, requiring specification of essential parameters and versions, such as file formats and other technical metadata. Atlases that have closed proprietary file formats may technically be digital, but without being fully machine accessible and interoperable, they are difficult to utilize in analytic tools and infrastructures.

At a *scholarly level*, anatomical parcellation and terminology should be comparable across atlases. The lack of consensus about terminologies, parcellation schemata, and boundary criteria among neuroanatomists is a major challenge for the development, use, and comparison of brain atlases^68–75^. Following different traditions, knowledge, and criteria, both domain experts and non-expert researchers may inevitably convey subjective and sometimes irreproducible information that is difficult to document. AtOM provides a foundation for organizing and communicating specific information about brain atlases in a standardized way that allows researchers to describe their interpretations more precisely, and thus contribute to increased reproducibility of results.

The value of interoperable atlases is substantial, allowing data integration, analysis and communication based on anatomical location. Brain atlases incorporated in various analytical tools open the possibility for efficient approaches to analyzing, sharing, and discovering data. For example, by analyzing images mapped to an atlas, the atlas information can be used to assign coordinates and terms to objects-of-interest^10,76^. Data from different publications analyzed with the same atlas are comparable, and data registered to the spatial module (reference data and coordinate system) of an atlas may also be re-analyzed with new or alternative annotation sets. Perhaps more importantly, by specifying the AtOM elements as standardized machine readable files, it becomes possible to incorporate different atlases as exchangeable modules in analytic tools and infrastructure systems^20–22,25,26^. Tools and systems using interoperable atlases can exploit the defined relations among the elements for automated operations, like data queries, calculations, or assignment of location identity to experimental data that have been associated with an atlas by spatial registration or semantic identification.

AtOM is used by multiple research and infrastructure groups, and is part of the Neuroscience Information Framework (RRID:SCR_002894), see Methods, and the openMINDS metadata framework for neuroscience graph databases (RRID:SCR_023173; https://github.com/HumanBrainProject/openMINDS). In particular, AtOM has served as base for the openMINDS SANDS extension (RRID:SCR_023498) which is focusing on the spatial anchoring of neuroscience data structures and includes the provision of controlled graph database descriptions for brain atlas and common coordinate spaces. openMINDS defines the semantic architecture of the EBRAINS Knowledge Graph. Other EBRAINS services, such as the EBRAINS Atlases (https://ebrains.hbp.eu/services/atlases) rely on openMINDS to robustly query for relevant data and correctly represent brain atlases and common coordinate spaces. The multilevel human brain atlas, an atlas framework that spans across multiple spatial scales and modalities hosted on the EBRAINS research infrastructure, exemplifies how several reference data, coordinate systems, and annotation set, developed over time, can be seamlessly incorporated, and presented to users through a single viewer tool. A growing repertoire of tools, services, and workflows within and outside of the EBRAINS research infrastructure rely on formal descriptions for automated incorporation of research products, including brain atlases and common coordinate spaces. AtOM provides a framework for keeping track of the complex relations among these resources and research products.

In conclusion, the primary value of AtOM is that it establishes a standardized framework for developers and researchers using brain atlases to create, use, and refer to specific atlas elements and versions. Atlas developers can use the model to create clearly citable and interoperable atlases. For developers incorporating atlases in tools, AtOM defines atlas elements as modules that can be seamlessly exchanged to accommodate atlases for other species or developmental stages, or to switch between versions, coordinate systems, or terminologies. By standardizing the communication and use of fundamental reference resources, we are convinced that AtOM will accelerate efficient analysis, sharing and reuse of neuroscience data.

## Methods

The first draft of AtOM (at the time called parcellation.ttl^77^) was developed by eliciting requirements and use cases from the Blue Brain Project (https://github.com/SciCrunch/NIF-Ontology/issues/49). To ingest atlas terminologies into the NIF standard ontology (RRID:SCR_005414) following AtOM, a python module (https://github.com/tgbugs/pyontutils/tree/master/nifstd/nifstd_tools/parcellation) was written to convert from a variety of formats into Web Ontology Language (OWL). An initial version of the core ontology and 24 atlas terminologies were created. These ontologies were loaded into SciGraph (RRID:SCR_017576; https://github.com/SciGraph/SciGraph) and queries (https://github.com/SciCrunch/sparc-curation/blob/67b534a939e2a271050c6edad97c707d8ec075d3/resources/scigraph/cypher-resources.yaml#L51-L267) were then written against the original data model using the Cypher query language to find atlases, terminologies, and individual terms for specific atlases, species, and developmental stages. These queries have been used in production systems for over 4 years. During this time additional atlases were ingested using the python module (now totaling 40) and an initial draft of the conceptual model for AtOM was developed (https://github.com/SciCrunch/NIF-Ontology/blob/master/docs/brain-regions.org). For a full record of the iterative development of the model to fully distinguish the major elements found in the current version (though not under their current names) see https://github.com/SciCrunch/NIF-Ontology/issues/49.

A second round of development involved further requirements collection in the context of atlas creation and the conceptual model was heavily revised, regularized, and extended in the context of the needs of the Human Brain Project (HBP) (https://github.com/SciCrunch/NIF-Ontology/commits/64c32abed9963073fab90dd5901d806fd8503da2 commit history from work during the HBP meeting in Oslo in November 21-22 2019) and the Allen Institute for Brain Sciences (https://github.com/SciCrunch/NIF-Ontology/commit/a40a8c786529f5b2e2a3a8007776d057c5830d2d, other interactions occurred, but do not have public records of their occurrence). Various iterations of the model were applied to a wide variety of atlases and atlas-like things, such as paper and digital atlases, ontologies, figures from publications, crudely drawn diagrams on table cloths, globes, geographic information systems, traditional cartographic maps, topological maps of the peripheral nervous system, and more. This was followed by collection of requirements and live ontology development carried out in the context of the HBP, which included alignment with the schemas of the openMINDS SANDS (RRID:SCR_023498) metadata model for reporting spatial metadata. The resulting ontological model was applied to a number of existing atlases, specifically the WHS rat brain atlas (RRID:SCR_017124)^14,38,39^, the AMBA CCF (RRID:SCR_020999) v3^3,13^, and the human Julich-Brain atlas (RRID:SCR_023277)^17,61^.

## Data Availability

AtOM (RRID:SCR_023499) is publicly available via GitHub: https://github.com/SciCrunch/NIF-Ontology/blob/atlas/ttl/atom.ttl. The ontology is available via BioPortal: http://purl.bioontology.org/ontology/ATOM. The 1.0 release of AtOM that corresponds to this paper is available via GitHub at https://github.com/SciCrunch/NIF-Ontology/releases/tag/atom-1.0.

## Code availability

Python code for generating parcellations for the NIF-Ontology is publicly available via GitHub: https://github.com/tgbugs/pyontutils/tree/master/nifstd/nifstd_tools/parcellation. Archives of release are available via Zenodo^77^.
